# Bacteria herald a new era of gene editing

**DOI:** 10.7554/eLife.00563

**Published:** 2013-01-29

**Authors:** David J Segal

**Affiliations:** 1 **David J Segal** is at the Genome Center and the Department of Biochemistry and Molecular Medicine, University of California, Davis, United Statesdjsegal@ucdavis.edu

**Keywords:** Cas9, endonuclease, genome editing, Human

## Abstract

The demonstration that nucleases guided by bacterial RNA can disrupt human genes represents a landmark in the rapidly developing field of genome engineering.

**Related research article** Jinek M, East A, Cheng A, Ma E, Doudna J. 2013. RNA-programmed genome editing in human cells. *eLife* **2**:e00471. doi: 10.7554/eLife.00471

**Image** The Cas9 protein could revolutionize genome engineering

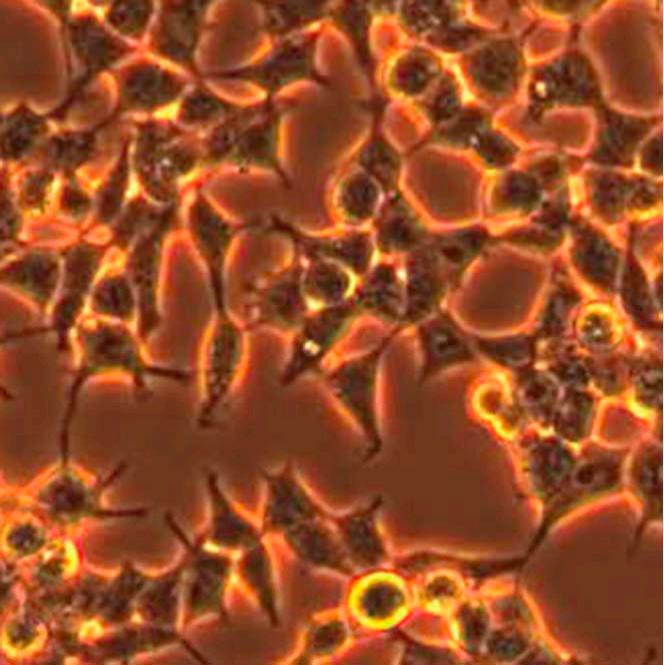

Tools for genome engineering seem to be improving faster than computers. Just over a year ago a set of gene editing techniques—zinc finger nucleases, transcription activator-like effector nucleases and engineered meganucleases—were chosen as the method of the year for 2011 by the journal *Nature Methods* (Baker, 2012). The work that laid the foundations for zinc finger nucleases was done about 20 years ago, but transcription activator-like effector (TALE) nucleases had only emerged in 2009. Then, at the end of 2012, TALE nucleases were selected as one of the 10 breakthroughs of the year by the journal *Science* (Alberts, 2012). Moreover, in an article entitled ‘Genomic cruise missiles’, *Science* predicted that a new genome engineering technique based on the bacterial protein Cas9—first reported in June 2012 (Jinek et al., 2012)—may well replace existing techniques. As a cluster of papers in *eLife* and elsewhere make clear, this prediction looks to be coming true (Cong et al., 2013; Mali et al., 2013; Jinek et al., 2013).

Zinc fingers are a type of protein that binds to DNA and they are found in about half of all transcription factors in the human genome. A zinc finger nuclease is made by attaching a nuclease—an enzyme that can cleave strands of DNA—to a zinc finger that has been re-engineered to bind to a particular DNA sequence (Perez-Pinera et al., 2012). Zinc finger nucleases can, therefore, make precise changes to the DNA of living cells by, for example, knocking out a gene, correcting a genetic mutation or, in the presence of appropriate donor DNA, inserting a new gene at a specific location.

**Figure 1.**
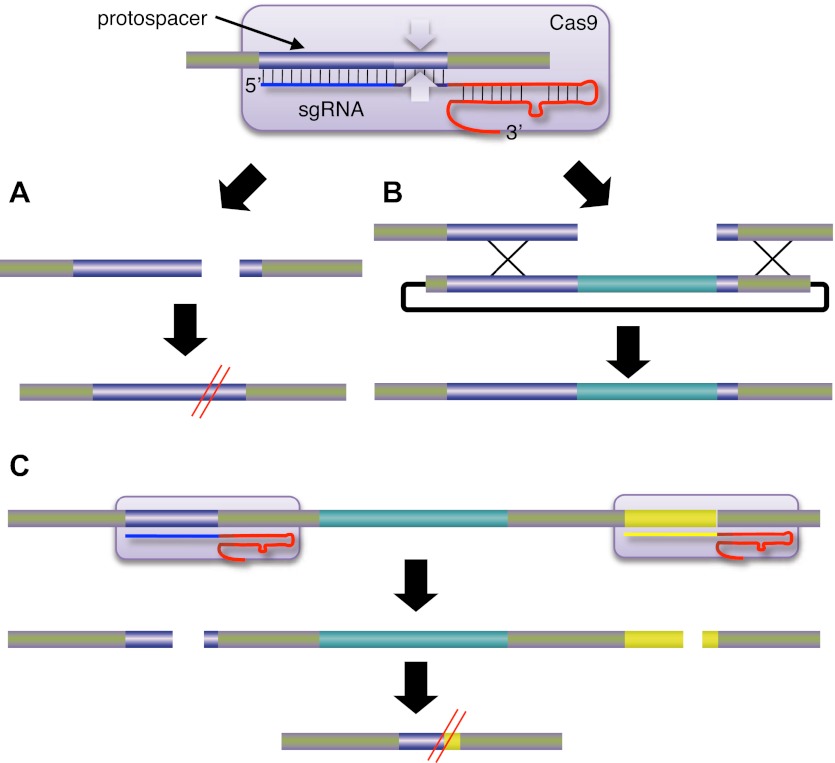
Jinek et al. attached a Cas9 nuclease (represented here by the purple rounded rectangle; the white arrows are the endonucleases) to a single-guide RNA (sgRNA), which guided it to a specific region of the target DNA called the protospacer. The sgRNA is a hybrid of two components: CRISPR RNA (crRNA; blue) and trans-activating crRNA (tracrRNA; red). (**A**) Cleavage of the target DNA by the Cas9 nuclease results in mutations that can knock out the target gene. (**B**) If an appropriate donor DNA molecule is available, genetic information (shown here in pale blue) can be added to the target DNA in a precise manner. (**C**) By targeting two Cas9 nucleases to different regions of the target DNA, it is possible to delete the genetic information between the two regions. CRISPR: clustered regularly interspaced short palindromic repeats; Cas: CRISPR-associated.

By the end of 2011, zinc finger nucleases had been used to knock out genes in rats, rabbits, and pigs, thus dethroning mice as the sole animal models of human genetics, and targeted gene disruptions had been performed on plants and zebrafish for the first time. Elsewhere, genetic manipulations of stem cells had created new avenues for disease research, and there were even zinc finger nucleases in clinical trials. Unfortunately, zinc finger nucleases were also difficult to make, and commercial sources were expensive. Moreover, although many sequences could be targeted, some could not. Finally, zinc finger nucleases sometimes cleaved DNA strands in the wrong place.

The paradigm for genome engineering shifted seemingly overnight in late 2009 with the discovery that TALEs—proteins produced by *Xanthamonas* bacteria to regulate transcription in their host plant cells—could bind to specific regions of DNA. The first TALE nuclease appeared in 2010, kits for their assembly appeared on the plasmid repository Addgene in 2011, and a method that can target almost 100 different genes with TALE nucleases was reported in April 2012 (Reyon et al., 2012). Compared to zinc finger nucleases, TALE nucleases are more accurate and can cleave a broader, seemingly comprehensive spectrum of DNA sequences, which is why today most experiments in genome engineering are performed with TALE nucleases.

Now, at the start of 2013, the paradigm seems set to shift again. Last year, a collaboration led by Jennifer Doudna of the University of California at Berkeley and Emmanuelle Charpentier of Umeå University sent shock waves through the genome engineering community by showing that a DNA nuclease called Cas9 could be targeted to specific DNA sequences if RNA was attached to it (Jinek et al., 2012). This new approach was based on the CRISPR/Cas system, which is part of the adaptive immune response of many bacteria and archaea. When a virus or plasmid invades a bacterium, segments of the invader's DNA are converted into CRISPR RNAs, or crRNA for short, by the immune response. This crRNA then associates with another type of RNA called tracrRNA to guide the Cas9 to a region called the ‘protospacer’ in the DNA of the invader. The Cas9 then cleaves the protospacer DNA on both strands (Figure 1). Importantly, Doudna, Charpentier and co-workers showed that the nuclease activity could be retargeted by simply designing a new crRNA. Moreover, this could be combined with the tracrRNA into one single-guide RNA.

Having demonstrated RNA-guided genome engineering in bacteria, the next challenge was to see if this approach would work in a eukaryotic nucleus. Now, in *eLife*, Doudna and co-workers—including Martin Jinek as first author—show that it can (Jinek et al., 2013). They do this by infecting human cells with two plasmids, one expressing the Cas9 protein, the other expressing single-guide RNA, and showing that this results in the cleavage of a particular gene. Such components will be significantly easier to make than TALE nucleases. For example, a typical TALE nuclease requires two new protein coding regions, each containing about 2000 base pairs, to be synthesized for each new target site, and the highest-throughput TALE assembly systems require large-scale material preparation and robotics for automation. In contrast, the Cas9 approach would require just one new RNA coding region of about 75 base pairs, and any investigator could easily order the hundreds or thousands of oligonucleotides needed for the experiments. Such ease of synthesis has enabled genome-wide screens of gene function using libraries of short hairpin RNA, so we can expect to see similar screens of thousands of genes with nucleases, possibly as soon as later this year.

Further support for this paradigm shift in genome engineering comes from papers by George Church of Harvard University and co-workers (Mali et al., 2013) and by Feng Zhang of the Broad Institute and co-workers (Cong et al., 2013). These groups demonstrated another advantage of CRISPR/Cas over TALE nucleases. Genetic deletions were produced by the simultaneous use of two crRNAs or single-guide RNA with Cas9, leading to contemporary double-strand breaks at distant sites and loss of the intervening DNA (Figure 1C). For TALE nucleases, such double cleavage events would require the synthesis of four new protein coding regions containing a total of about 8000 base pairs. These two studies also extended the Cas9 approach to human induced pluripotent stem cells and mouse cell lines, and demonstrated alterations by both homologous recombination and non-homologous end joining mechanisms. In general, CRISPR/Cas systems were found to be comparable to zinc finger and TALE nucleases in terms of activity, or to be more active.

Many important questions still remain, such as the extent of ‘off-target’ events. Moreover, it seems that as few as 14–16 base pairs of DNA are actually specified by CRISPR/Cas systems, which is unlikely to be sufficient to define a unique address in a human genome. However, the new approach will be tested and improved at a furious pace in the coming months, and the Cas9 approach may well supplant TALEs as the nuclease of choice by the summer, unless there is another paradigm shift before then.
